# P-824. A Tertiary Care Hospital's Eight-Year Experience with *Acinetobacter* Bloodstream Infections in the Pediatric Population

**DOI:** 10.1093/ofid/ofae631.1016

**Published:** 2025-01-29

**Authors:** Ece Erci, Gulhadiye Avcu, Nimet Melis Bilen, Sema Yıldırım Arslan, Gizem Guner, Pınar Yazıcı Özkaya, Feriha Cilli, Zumrut Sahbudak Bal

**Affiliations:** Medical School of Ege University, Izmir, Izmir, Turkey; Ege university, pediatric infectious diseases, izmir, Izmir, Turkey; Division of Infectious Disease, Department of Pediatrics, BORNOVA, Izmir, Turkey; Division of Infectious Disease, Department of Pediatrics, BORNOVA, Izmir, Turkey; Medical School of Ege University,Division of Infectious Disease, Department of Pediatrics, Bornova, Izmir, Turkey; Medical School of Ege University, Division of Pediatric Intensive Care Unit, Department of Pediatrics, bornova, Izmir, Turkey; Medical Microbiology, Bornova, Izmir, Turkey; Medical School of Ege University,Division of Infectious Disease, Department of Pediatrics, Bornova, Izmir, Turkey

## Abstract

**Background:**

*Acinetobacter spp* has emerged as an important nosocomial pathogen causing life-threatening infections in hospitalized patients. Since carbapenem-resistant *Acinetobacter* bloodstream infections have been increasingly reported in the pediatric population worldwide in recent years, we aimed to evaluate the clinical characteristics and identify possible risk factors in hospitalized patients with carbapenem-resistant bloodstream infections.

**Methods:**

We retrospectively analyzed the results of blood-stream infections caused by *Acinetobacter spp*. in hospitalized children between 2014-2022. Demographic features and clinical and laboratory findings were evaluated.

**Results:**

Bloodstream infections due to *Acinetobacter* spp. were detected in 70 hospitalized children. Carbapenem resistance was present in 38 (54.2%) of the patients. The mean age of patients with carbapenem-resistant (CR) *Acinetobacter* bloodstream infection (21 males and 17 females) was 5.7±0.95 years and 3.9±0.74 years in the carbapenem-susceptible (CS) group (13 males and 19 females) (p< 0.005). The most common pathogen in the CR group (97.4%) and CS group (56.3%) was *Acinetobacter baumannii*. While 15.3% of the CR patients were previously healthy, 3.1% of the CS group had no history of previous diseases. The most important predisposing risk factors for the development of carbapenem resistance were invasive mechanical ventilation (p< 0.005), previous antibiotic use (p< 0.005), and history of meropenem use (p< 0.005). Combination therapy was initiated in 64.6% of patients in the CR group and 53.1% in the CS group. The most frequently used antibiotic regimens in the CR group were meropenem-colistin (15.8%), colistin-amikacin (10.5%), and colistin-sulfamethoxazole and trimethoprim (SMZ-TMP) (7.9%). The mean treatment duration for the groups was 13.3±0.98 days for the CR group and 11.3±1 days for the CS group (p:0.523). The infection-related mortality rates for the CR and the CS groups were 36.8% and 21.9%, respectively, and there was no statistically significant difference between the groups (p:0.136).

**Conclusion:**

Our single-center experience aims to emphasize the importance of rational antibiotic use and enhance the awareness of clinicians about the increasing carbapenem resistance.

**Disclosures:**

**All Authors**: No reported disclosures

